# The Asymptotics of Recovery Probability in the Dual Renewal Risk Model with Constant Interest and Debit Force

**DOI:** 10.1155/2015/504987

**Published:** 2015-03-22

**Authors:** Hao Wang, Lin Xu

**Affiliations:** Department of Statistics, Anhui Normal University, Wuhu, Anhui 241002, China

## Abstract

The asymptotic behavior of the recovery probability for the dual renewal risk model with constant interest and debit force is studied. By means the idea of Markov Skeleton method, we studied the times that the random premium incomes happened and transformed the continuous time model into a discrete time model. By investigating the fluctuations of this discrete time model, we obtained the asymptotic behavior when the random premium income belongs to a kind of heavy-tailed distributions.

## 1. Introduction

The classical risk model is specified as

(1) $$\begin{array}{c} U\left(t\right)=x+ct-S\left(t\right), \end{array}$$

where *x* ≥ 0 is the initial surplus and *c* is the constant rate at which the premiums are received. The aggregate claims process {*S*(*t*)} is assumed to be a compound Poisson process, which denotes the total number of claims up to time *t*. Denote the time of arrival of the *i*_th_ claim by *T*_*i*_ and the size of the *i*_th_ claim by *Y*_*i*_. More details about the surplus process can be found in Asmussen and Albrecher [1] and Rolski et al. [2]. As pointed out by Albrecher et al. [3], its dual process may also be relevant for companies whose inherent business involves a constant flow of expenses while revenues arrive occasionally due to some contingent events (e.g., discoveries and sales). For instance, pharmaceutical or petroleum companies are prime examples of companies for which it is reasonable to model their surplus process as

(2) $$\begin{array}{c} U_{t}=x-ct+\overset{N_{t}}{\underset{i=1}{\sum }}X_{k}. \end{array}$$

Here, *x* is again the initial surplus, but the constant *c* is now the rate of expenses, assumed to be deterministic and fixed. The aggregate claims process *S*(*t*) = ∑_*k*=1_^*N*_*t*_^ *X*_*k*_ is assumed to be a compound renewal process. The negative claim sequence {*X*_*k*_, *k* = 1,2, 3,…} is assumed to be a sequence of independent and identically distributed (i.i.d.) nonnegative random variables (r.v.s.) with common distribution function $F=1-\bar{F}$; we said $\bar{F}$ is the tailed distribution. The interoccurrence times {*θ*_*k*_, *k* = 1,2, 3,…} form another sequence of i.i.d. positive random variables. {*θ*_*k*_, *k* = 1,2, 3,…} and {*X*_*k*_, *k* = 1,2, 3,…} are mutually independent. The occurrence times of the successive claims, *T*_*n*_ = ∑_*k*=1_^*n*^ *θ*_*k*_, *n* = 1,2, 3,…, constitute a renewal counting process

(3) $$\begin{array}{c} N_{t}=♯\left\{n=1,2,3,\ldots :T_{n}\leq t\right\},\;t\geq 0. \end{array}$$

The past decade has witnessed an increasing attention on the research of dual risk model. For example, see Albrecher et al. [3] for optimal dividend problem, see Cheung and Drekic [4] for dividend approximation and dual risk model with perturbation, and see Yao et al. [5] for optimal dividend and equity issuance. Due to the positive safety loading, in dual risk model, when the operation time scale is infinite, the ruin probability of dual risk model is zero; thus, it is meaningless to discuss the ruin problem under dual risk model. However, there is a constant consumption in the dual risk model; thus, it has occasionally happened that the surplus of dual risk model is negative; in this case, the decision maker will care the probability that the surplus of the model became positive; we define this probability as the “recovery probability.” This conception is of both theoretical value and practical relevance. In this paper, we focus on the asymptotic behavior of the recovery probability under the dual renewal risk model, which covers the compound Poisson dual risk model. The rest of this paper is organized as follows. In Section 2, we present an introduction to the dual renewal risk model with constant interest force and debit interest force and the problem to be investigated.  Section 3 provides the main results and the corresponding proofs.

## 2. Model and Problem

In this section, we consider the case that the research institution would like to invest his surplus in bond market or borrow money from the bank; both the investment interest force or the debit interest force are the same, say *δ* > 0. If there are no claims in the interval (0, Δ*t*), then the surplus up to time *t* is given by

(4) $$\begin{array}{c} U\left(t+\Delta t\right)=U\left(t\right)-c\Delta t+U\left(t\right)\left(e^{\delta \Delta t}-1\right); \end{array}$$

letting Δ*t* → 0, we can obtain

(5) $$\begin{array}{c} U^{\prime }\left(t\right)=-c+\delta U\left(t\right). \end{array}$$

It is easy to see, if *U*(*t*) > *c*/*δ*, then *U*′(*t*) > 0; the surplus is increased and is not less than *c*/*δ*; if 0 < *U*(*t*) < *c*/*δ*, then *U*′(*t*) < 0; the surplus is decreased and the insurance company may be ruin; if *U*(*t*) < 0, we know it is possible that the surplus *U*(*t*) can recover to *c*/*δ*. Above all, we can define the absolute positive profit as

(6) $$\begin{array}{c} U\left(t\right)>\frac{c}{\delta }. \end{array}$$

In this paper, we will discuss the recovery probability which is the probability of the surplus recover to *c*/*δ* when *U*(*t*) ≤ 0.

Now we let *δ* > 0 be the constant force of interest so that after time *t* a capital *x* becomes *xe* ^*δt*^. Then, the total surplus which is denoted by *W*_*δ*_(*t*) is given by

(7) $$\begin{array}{c} W_{\delta }\left(t\right)=xe^{\delta t}-c\overset{t}{\underset{0}{\int }}e^{\delta (t-y)}+\overset{N_{t}}{\underset{k=1}{\sum }}X_{k}e^{\delta (t-T_{k})},\;t\geq 0. \end{array}$$

Now, we can define the finite-time recovery probability as

(8) $$\begin{array}{c} \psi \left(x,t\right)=P\left(\underset{0\leq s\leq t}{\sup}W_{\delta }\left(s\right)>\frac{c}{\delta }\;|\;W_{\delta }\left(0\right)=x\right),\;x\leq 0,\;\;t\geq 0. \end{array}$$

At occurrence time *T*_*n*_ = ∑_*k*=1_^*n*^ *θ*_*k*_, we observe the value *W*_*δ*_(*T*_*n*_) which represents the surplus immediately after paying the *n*th claim, *n* = 1,2,…. By virtue of (7), we have

(9) WδTn=xeδTn−c∫0TneδTn−ydy+∑k=1nXkeδTn−Ty=xeδTn−1·eδθn−c∫0Tn−1eδTn−ydy −c∫Tn−1TneδTn−ydy+∑k=1n−1XkeδTn−Ty+Xn=Wδ(Tn−1)eδθn−cδ(eδθn−1)+Xn.

Let

(10) $$\begin{array}{c} V_{n}=W_{\delta }\left(T_{n}\right)-\frac{c}{\delta },\;n=1,2,\ldots ; \end{array}$$

it follows that

(11) $$\begin{array}{c} V_{0}=W_{\delta }\left(T_{0}\right)-\frac{c}{\delta }=x-\frac{c}{\delta },\\ V_{n}=V_{n-1}e^{\delta \theta_{n}}+X_{n},\;n=1,2,\ldots ,\\ V_{n}=\left(x-\frac{c}{\delta }\right)\overset{n}{\underset{k=1}{\prod }}e^{\delta \theta_{k}}+\overset{n}{\underset{k=1}{\sum }}X_{k}\overset{n}{\underset{i=k+1}{\prod }}e^{\delta \theta_{i}},\;n=1,2,\ldots \end{array}$$

Since recovery can happen only at the time of a claim occurrence, we rewrite the finite-time recovery probability in (8) as

(12) ψx,t=Psup⁡0≤s≤tWδ(s)>cδ ∣ Wδ(0)=x=Psup⁡1≤n≤NtVn>0 ∣ V0=x−cδ=Psup⁡1≤n≤Ntx−cδ+∑k=1nXk∏i=1kYi>0.

Since sup⁡_1≤*n*≤*N*_*t*__{∑_*k*=1_^*n*^ *X*_*k*_∏_*i*=1_^*k*^ *Y*_*i*_} is increased, let *Y*_*i*_ = *e* ^−*δθ*_*i*_^; we obtain

(13) $$\begin{array}{c} \psi \left(x,t\right)=P\left(\overset{N_{t}}{\underset{k=1}{\sum }}X_{k}\overset{k}{\underset{i=1}{\prod }}Y_{i}>\frac{c}{\delta }-x\right),\;x\leq 0,\;\;t\geq 0; \end{array}$$

here, ∏_*i*=0_^*k*^ *Y*_*i*_ = *e* ^−*δT*_*k*_^ ∈ [0,1]. So we can study the recovery probability in (8) by the (13).

Similarly, for the infinite-time recovery probability defined in (13), we have

(14) $$\begin{array}{c} \psi \left(x,\infty \right)=P\left(\overset{\infty }{\underset{k=1}{\sum }}X_{k}\overset{k}{\underset{i=1}{\prod }}Y_{i}>\frac{c}{\delta }-x\right),\;x\leq 0,\;\;t\geq 0. \end{array}$$

Here and henceforth, all limit relationships are for *x* → *∞* unless stated otherwise.

For two positive functions *f*(*x*) and *g*(*x*), the relation *f*(*x*) ~ *g*(*x*) amounts to the conjunction of the relations limsup⁡*f*(*x*)/*g*(*x*) ≤ 1 and liminf⁡*f*(*x*)/*g*(*x*) ≥ 1, which are denoted as *f*(*x*)≲*g*(*x*) and *f*(*x*) ≳ *g*(*x*), respectively. Next, we will define the convolution. For two distributions *F*_1_ and *F*_2_ on [0, *∞*), we can say *F*_1_∗*F*_2_ is their convolution; that is,

(15) $$\begin{array}{c} F_{1}*F_{2}\left(x\right)=\overset{x}{\underset{0-}{\int }}F_{1}\left(x-y\right)F_{2}dy,\;x\geq 0. \end{array}$$

Furthermore, we write *F* ^1∗^ = *F* and *F* ^*n*∗^ = *F* ^(*n*−1)∗^∗*F* for every *n* = 2,3,….

For the general renewal risk model, an asymptotic expression for the finite time ruin probability is presented in [6]; that is,

(16) $$\begin{array}{c} \psi \left(x,t\right)\sim \lambda \exp\left\{\frac{\lambda }{\delta }\overset{\gamma }{\underset{\gamma e^{-\delta t}}{\int }}\frac{Ee^{sX}-1}{s}\;ds-\frac{\gamma c}{\delta }\right\}\overset{t}{\underset{0}{\int }}\bar{F}\left(xe^{\delta s}\right)ds. \end{array}$$

In recent years, with the study of classical model and the development of the financial and insurance business, more and more people are interested in dual risk model; however, an analogue problem in insurance company is the dividend payment or high gain tax payment. Thus, the problem studied here sounds reasonable.

## 3. Main Results

In order to complete our results, we should have some definitions and lemmas.

**Definition 1 .**

A distribution *F* on [0, *∞*) is said to belong to the class *𝔍* if

(17) $$\begin{array}{c} \underset{x\to \infty }{\lim}\frac{\bar{F^{n*}}\left(x\right)}{\bar{F}\left(x\right)}=n, \end{array}$$

for every *n* ≥ 2; that is, *F* ∈ *𝔍*.

**Definition 2 .**

A distribution *F* on [0, *∞*) is said to belong to the class *𝒮*(*γ*) for some *γ* ≥ 0 if

(18) $$\begin{array}{c} \underset{x\to \infty }{\lim}\frac{\bar{F}\left(x-y\right)}{\bar{F}\left(x\right)}=e^{\gamma y}, \end{array}$$

for every real number *y* and the limit

(19) $$\begin{array}{c} \underset{x\to \infty }{\lim}\frac{\bar{F^{2*}}\left(x\right)}{\bar{F}\left(x\right)}=2\overset{\infty }{\underset{0-}{\int }}e^{\gamma y}F\left(dy\right) \end{array}$$

exists and is finite; that is, *F* ∈ *𝒮*(*γ*). A larger class, *ℒ*(*γ*), is defined by relation (18) alone.

A distribution function *F* concentrated on (−*∞*, *∞*) is still said to be subexponential to the right if *F* ^+^(*x*) = *F*(*x*)1_(0≤*x*<*∞*)_ is subexponential, and we usually denote by *𝒮* the subexponential classes; see for example, [7]. Since it was introduced by [8–10], the subexponential class *𝒮* has been extensively investigated by many researchers and applied to various fields. This class is often used to model claim-size distributions; see, for example, [11–13].

**Definition 3 .**

A distribution *F* on [0, *∞*) is said to belong to *ℛ*_−*∞*_ if

(20) $$\begin{array}{c} \underset{x\to \infty }{\lim}\frac{\bar{F}\left(xy\right)}{\bar{F}\left(x\right)}=0, \end{array}$$

for arbitrary *y* > 1.

**Lemma 4 .**

Let *F*, *F*_1_, and *F*_2_ be three distributions on [0, *∞*) such that *F* ∈ *𝒮*(*γ*) and that the $\lim l_{i}=\lim_{x\to \infty }(\bar{F_{i}}\left(x\right)/\bar{F}\left(x\right))$ exists and is finite for *i* = 1,2. Then,

(21) $$\begin{array}{c} \underset{x\to \infty }{\lim}\frac{\bar{F_{1}*F_{2}}\left(x\right)}{\bar{F}\left(x\right)}=l_{1}\overset{\infty }{\underset{0-}{\int }}e^{\gamma y}F_{2}\left(dy\right)+l_{2}\overset{\infty }{\underset{0-}{\int }}e^{\gamma y}F_{1}\left(dy\right). \end{array}$$

**Proof**

See [14], Proposition 2.

**Lemma 5 .**

Let *F*_1_ and *F*_2_ be two distributions on [0, *∞*). If *F*_1_ ∈ *𝒮*(*γ*), *F*_2_ ∈ *ℒ*(*γ*), and $\bar{F_{2}}(x)=\circ (\bar{F_{1}}(x))$, then *F*_1_∗*F*_2_ ∈ *𝒮*(*γ*) and

(22) $$\begin{array}{c} \bar{F_{1}*F_{2}}\left(x\right)\sim \bar{F_{1}}\left(x\right)\overset{\infty }{\underset{0-}{\int }}e^{\gamma y}F_{2}\left(dy\right)+\bar{F_{2}}\left(x\right)\overset{\infty }{\underset{0-}{\int }}e^{\gamma y}F_{1}\left(dy\right). \end{array}$$

**Proof**

See [6], Corollary 1.

**Lemma 6 .**

Let *F* be a distribution on [0, *∞*). If *F* ∈ *𝒮*(*γ*), then it holds for each fixed *n* = 1,2,… that

(23) $$\begin{array}{c} \bar{F^{n*}}\left(x\right)\sim n{\left(\overset{\infty }{\underset{0-}{\int }}e^{\gamma y}F(dy)\right)}^{n-1}\bar{F}\left(x\right). \end{array}$$

**Proof**

See [10], page 665.

**Lemma 7 .**

Let {*X*_*k*_, *k* = 1,2, 3,…} be a sequence of independent and identically distributed (i.i.d.) nonnegative random variables with common distribution function *F*, *F* ∈ *𝔍*. {*σ*_*k*_, *k* = 1,2,…} is a sequence of nonnegative random variables, {*σ*_*k*_, *k* = 1,2,…} and {*X*_*k*_, *k* = 1,2,…} are mutually independent, and if for some 0 < *a* ≤ *b* < *∞* and all 1 ≤ *k* ≤ *n*, satisfies

(24) $$\begin{array}{c} P\left(a\leq \sigma_{k}\leq b\right)=1, \end{array}$$

then

(25) $$\begin{array}{c} P\left(\overset{n}{\underset{k=1}{\sum }}\sigma_{k}X_{k}>x\right)\sim \overset{n}{\underset{k=1}{\sum }}P\left(\sigma_{k}X_{k}>x\right). \end{array}$$

**Proof**

See [7], Proposition 5.1.

**Lemma 8 .**

Let {*N*_*t*_, *t* ≥ 0} be a renewal process. The interoccurrence time {*θ*_*k*_, *k* ≥ 1} forms another sequence of i.i.d. positive random variable with common distribution function *H*; it is easy to see that *H*_*n*_ which is the distribution of the *n*th claim occurrence time *T*_*n*_ = ∑_*k*=1_^*n*^ *θ*_*k*_ is the convolution of *H*. If *m*(*t*) = *𝔼N*_*t*_ is the renewal function, then we have

(26) $$\begin{array}{c} m\left(t\right)=\overset{\infty }{\underset{n=1}{\sum }}H_{n}\left(t\right). \end{array}$$

**Proof**

See [15], page 49.

### 3.1. Main Results and Proof

**Theorem 9 .**

In the dual renewal risk model with constant force of interest *δ* > 0, the number of claims {*N*_*t*_, *t* ≥ 0} is a renewal process, *m*(*t*) is the renewal function, and the claim sizes {*X*_*k*_, *k* ≥ 1} and {*N*_*t*_, *t* ≥ 1} are mutually independent. If *F* ∈ *𝔍*∩*𝒮*(*γ*), then

(27) $$\begin{array}{c} \psi \left(x,t\right)\sim \overset{t}{\underset{0}{\int }}e^{-\gamma \left(c/\delta \right)e^{\delta y}}P\left(Xe^{-\delta y}>-x\right)dm\left(y\right),\;x\leq 0, \end{array}$$

for arbitrary *t* > 0.

**Proof**

Starting with (13) and conditioning on *N*_*t*_, we have

(28) ψx,t=P∑k=1NtXk∏i=1kYi>cδ−x=∑n=1∞P∑k=1NtXk∏i=1kYi>cδ−x ∣ Nt=nP(Nt=n).

Since ∏_*i*=1_^*k*^ *Y*_*i*_ = *e* ^−*δT*_*k*_^ ∈ [*e* ^−*δt*^, 1] for every 1 ≤ *k* ≤ *n*, by Lemma 7, we have

(29) ψx,t~∑n=1∞ ∑k=1nPXke−δTk>cδ−xPNt=n=∑n=1∞ ∑k=1nPXke−δTk>cδ−x,Nt=n.

We know {*N*_*t*_ ≥ *n*}⇔*T*_*n*_ ≤ *t*; then,

(30) ψx,t~∑k=1∞ ∑n=knPXke−δTk>cδ−x,Nt=n=∑k=1∞PXke−δTk>cδ−x,Nt≥k=∑k=1∞PXke−δTk>cδ−x,Tk<t=∑k=1∞∫0tPXe−δy>cδ−xdHky=∫0tPXe−δy>cδ−xd∑k=1∞Hky=∫0tPXe−δy>cδ−xdmy;

since *F* ∈ *𝒮*(*γ*), then

(31) $$\begin{array}{c} \frac{\lim_{x\to \infty }P\left(X>\left(c/\delta \right)e^{\delta y}-xe^{\delta y}\right)}{P\left(X>-xe^{\delta y}\right)}=e^{-\gamma (c/\delta )e^{\delta y}}. \end{array}$$

So we have

(32) $$\begin{array}{c} \psi \left(x,t\right)\sim \overset{t}{\underset{0}{\int }}e^{-\gamma (c/\delta )e^{\delta y}}P\left(Xe^{-\delta y}>-x\right)dm\left(y\right). \end{array}$$

We present an asymptotic expression for the finite-time recovery probability in Theorem 9. In the following, we will show the last main result of the paper.

**Theorem 10 .**

In the dual renewal risk model with constant force of interest *δ* > 0, if *F* ∈ *𝒮*∩*ℛ*_−*∞*_ for some *γ* ≥ 0, *x* ≤ 0, then

(33) $$\begin{array}{c} Ee^{\gamma S_{\infty }}<\infty ,\;where\;S_{\infty }=\overset{\infty }{\underset{k=1}{\sum }}X_{k}\overset{k}{\underset{i=1}{\prod }}Y_{i}, \end{array}$$

(34) $$\begin{array}{c} \psi \left(x,\infty \right)\sim Ee^{\gamma S_{\infty }}P\left(X_{1}Y_{1}>\frac{c}{\delta }-x\right),\;x\leq 0, \end{array}$$

where {*Y*_*i*_ = *e* ^−*δθ*_*i*_^, *i* = 1,2,…} is a sequence of i.i.d. positive random variables with common distribution function *G*.

**Proof**

The proof of *𝔼e* ^*γS*_*∞*_^ < *∞* is the same as the one for Theorem 3.2 of [16]; we do not copy the steps here. Next, we will prove (34). Let ${\tilde{S}}_{\infty }$ be a copy of *S*_*∞*_ independent of {(*X*_*k*_, *Y*_*k*_), *k* = 1,2,…}. Then, for every *n* = 1,2,…,

(35) $$\begin{array}{c} S_{\infty }≜S_{T_{n}}+{\tilde{S}}_{\infty }\overset{n}{\underset{i=1}{\prod }}Y_{i}. \end{array}$$

Therefore,

(36) $$\begin{array}{c} S_{\infty }\geq S_{T_{n}}, \end{array}$$

(37) $$\begin{array}{c} S_{\infty }\leq S_{T_{n}}+X^{*}\overset{n}{\underset{i=1}{\prod }}Y_{i}; \end{array}$$

here *X* ^*^ = (*Z*∣*Z* > *c*/*δ* − *x*_0_) is a new conditional random variable, and the distribution still belongs to the intersection *𝒮*(*γ*)∩*ℛ*_−*∞*_; *x*_0_ < 0 is a small enough constant.

So from (36) and according to the (13), we can obtain

(38) PS∞>cδ−x ≥PSTn>cδ−x =P∑k=1NtXk∏i=1kYi>cδ−x =PX1Y1+∑k=2NtXk∏i=2kYi>cδ−x =PX1+∑k=2NtXk∏i=2kYiY1>cδ−x =∫01PX1+∑k=2NtXk∏i=2kYi>c/δ−xyG(dy).

Since

(39) P∑k=2NtXk∏i=2kYi>cδ−x ≤P∑k=2NtXkY1>cδ−x =∫01P∑k=2NtXk>c/δ−xyG(dy)

and the Lemma 6, we can see

(40) P∑k=2NtXk>c/δ−xy ~Nt−1∫0−∞eγyFdyNt−2F¯c/δ−xy =Nt−1EeγXNt−2F¯c/δ−xy.

We know *F* ∈ *ℛ*_−*∞*_, so $\lim_{x\to \infty }(\bar{F}\left(xy\right)/\bar{F}\left(x\right))=0$, *y* > 1, that is, $\bar{F}(xy)=\circ (\bar{F}(x))$, we can obtain $\bar{F}(-x/y)=\circ (\bar{F}(-x))$. So

(41) P∑k=2NtXk∏i=2kYi>cδ−x ≲(Nt−1)EeγXNt−2F¯c/δ−xy =∘F¯cδ−x;

we deduce that

(42) $$\begin{array}{c} \frac{\lim_{x\to \infty }P\left(\sum_{k=2}^{N_{t}}X_{k}\prod_{i=2}^{k}Y_{i}>c/\delta -x\right)}{\bar{F}\left(c/\delta -x\right)}=0,\\ \frac{\lim_{x\to \infty }P\left(X_{1}>c/\delta -x\right)}{\bar{F}\left(c/\delta -x\right)}=1. \end{array}$$

By Lemma 4, we obtain that

(43) PX1+∑k=2NtXk∏i=2kYi>c/δ−xy ~1·∫0−∞eγyF2dy+0·∫0−∞eγyF1dy  ·F¯c/δ−xy =∫0−∞eγyF2dyF¯c/δ−xy =Eeγ∑k=2NtXk∏i=2kYiF¯c/δ−xy,

so

(44) PS∞>cδ−x ≥Eeγ∑k=2NtXk∏i=2KYi∫01F¯c/δ−xyGdy =Eeγ∑k=2NtXk∏i=2KYiPX1Y1>cδ−x.

Then,

(45) $$\begin{array}{c} \underset{x\to \infty }{\mathrm{liminf}}\frac{P\left(S_{\infty }>c/\delta -x\right)}{P\left(X_{1}Y_{1}>c/\delta -x\right)}\geq Ee^{\gamma (\sum_{k=2}^{N_{t}}X_{k}\prod_{i=2}^{k}Y_{i})}. \end{array}$$

Clearly, ∑_*k*=2_^*N*_*t*_^ *X*_*k*_∏_*i*=2_^*k*^ *Y*_*i*_ converges to *S*_*∞*_ in distribution as *n* → *∞*. Therefore, by the dominated convergence theorem, the expectation on the right-hand side above converges to *𝔼e* ^*γS*_*∞*_^ as *n* → *∞*; that is,

(46) $$\begin{array}{c} \underset{x\to \infty }{\mathrm{liminf}}\frac{P\left(S_{\infty }>c/\delta -x\right)}{P\left(X_{1}Y_{1}>c/\delta -x\right)}\geq Ee^{\gamma S_{\infty }}. \end{array}$$

It is easy to construct the corresponding asymptotic upper bound by (37). Similarly as above (by Lemmas 4 and 5),

(47) $$\begin{array}{c} \underset{x\to \infty }{\mathrm{limsup}}\frac{P\left(S_{\infty }>c/\delta -x\right)}{P\left(X_{1}Y_{1}>c/\delta -x\right)}\leq Ee^{\gamma (\sum_{k=2}^{N_{t}}X_{k}\prod_{i=2}^{k}Y_{i}+X^{*}\prod_{i=2}^{n}Y_{i})}. \end{array}$$

Clearly, ∑_*k*=2_^*N*_*t*_^ *X*_*k*_∏_*i*=2_^*k*^ *Y*_*i*_ + *X* ^*^∏_*i*=2_^*n*^ *Y*_*i*_ converges to *S*_*∞*_ in distribution as *n* → *∞*. Therefore, similarly as above,

(48) $$\begin{array}{c} \underset{x\to \infty }{\mathrm{limsup}}\frac{P\left(S_{\infty }>c/\delta -x\right)}{P\left(X_{1}Y_{1}>c/\delta -x\right)}\leq Ee^{\gamma S_{\infty }}. \end{array}$$

This completes the proof.
